# Balancing between dual belongings when organised into interdisciplinary teams, with the trust model as the context: A qualitative study

**DOI:** 10.1186/s12875-024-02554-7

**Published:** 2024-08-24

**Authors:** Ruth-Ellen Slåtsveen, Torunn Wibe, Liv Halvorsrud, Anne Lund

**Affiliations:** 1 OsloMet- Oslo Metropolitan University, Oslo, Norway; 2 Centre for Development of Institutional and Home Care Services in Oslo, Oslo, Norway

**Keywords:** Health professions, Home-based healthcare, Interdisciplinary teams, Primary care, Trust model

## Abstract

**Background:**

Home-based healthcare services are facing challenges and pressures of increasing needs due to an ageing population, rising workload for an overburdened workforce, and limited financial resources. The trust model is an approach to address the challenges, by organizing the home-based healthcare services into smaller, autonomous interdisciplinary teams. The aim is to involve users and next of kin in decision-making and trusting frontline workers’ professional judgement, thus making the services more flexible and individually tailored. This study explores frontline workers’ practices and experiences of working within interdisciplinary teams according to the trust model’s goals.

**Methods:**

Observations, individual-, and focus groups interviews were conducted within home-based healthcare service in a Norwegian municipality. The participants were leaders and frontline workers at different levels of the home-based healthcare services, including registered nurses, auxiliary nurses, occupational therapists, physiotherapists, and other unskilled healthcare personnel. Data was analysed thematically.

**Results:**

The results are presented in terms of themes: ‘We all want the best for service users’, ‘Belonging to an interdisciplinary team’ and ‘Maintaining belonging to those with similar work tasks and responsibilities’. The results show a diversity among the participants’ experiences of working within interdisciplinary teams. It demonstrates a dilemma between creating belonging to and forming identities within the interdisciplinary team, and at the same time, the importance of maintaining belonging and identity with those in the same profession or with the same tasks and responsibilities.

**Conclusion:**

This study suggests that the frontline workers need for dual belonging seems to be underestimated within the trust model, and by acknowledging this, organisations and policymakers can create environments that support both. Which in turn can enhance the possibility to deliver flexible and individually tailored services for service users.

**Supplementary Information:**

The online version contains supplementary material available at 10.1186/s12875-024-02554-7.

## Background

Globally, home-based healthcare services are under unprecedented pressure due to the increased needs of an ageing population. They are also facing challenges such as rising workload of an overburdened workforce and limited financial resources [1–3]. Additionally, current healthcare delivery and organisational models are perceived as fragmented and hierarchical [4]. Studies show that frontline workers are grappling with the dichotomy between service delivery and adhering to organisational policies which results in inflexible services lacking personal continuity, with limited opportunities for ongoing adjustment based on the service user’s needs [5–8] Aiming to address these challenges, international and national healthcare policies, and guidelines have been endorsing the demand for new ways of working, service reorganisation, more interdisciplinary teamwork and the application of digital technologies in health care [3, 9]. This paper elaborates on the trust model, which is one approach used in Norway to meet the necessity for new ways of working in home-based healthcare services. The trust model encourages decision-making within smaller, autonomous interdisciplinary teams consisting of occupational therapists, physiotherapists, registered nurses, auxiliary nurses, case managers (former purchaser unit employees) and other unskilled healthcare personnel, hereafter referred to as frontline workers (FLWs). The model’s primary objective is to enhance the involvement of both service users and their next of kin in the decision-making process. Furthermore, it seeks to empower FLWs with the authority to make decisions and tailor the services they offer according to individual needs based on the users’ health conditions, enabling them to live their lives at home as long as possible and perform the activities they find useful. Hence, trust should be placed in the professional assessments made by FLWs. This approach is meant to increase the flexibility and individual tailoring of home-based healthcare services [7, 8]. Another objective of a team-based organisation is grounded in the understanding that interdisciplinary collaboration can produce results that surpass those achieved by an individual FLW. However, this requires teams to be able to mobilise and coordinate FLWs’ resources and create team identities and social belonging [10].

It seems that earlier research on the trust model focused on intervention conditions, the leader’s role, FLWs’ autonomy and how the model is understood, implemented, and experienced. The Norwegian trust model was inspired by the Dutch Buurtzorg model, which has inspired other countries to adopt similar models, although these models do not have an interdisciplinary focus [2, 8, 11–14]. There have been some similar findings in the research on the different model versions. Limited resources, lack of competence, communication barriers and old habits have been identified as making it difficult to change the institutional patterns in an organisation [7, 8, 11]. Studies of the trust model have indicated positive outcomes through increased interdisciplinary collaboration, flexibility in decision-making and personal continuity [7, 8].

In our study, a needs-led research process was applied to develop research questions with FLWs and users on how the trust model affects service design and how it is practiced within home-based healthcare services [15]. Other studies have demonstrated the importance of understanding the context of collaboration without focusing on single cases or coming from the perspective of a single profession [16, 17]. Interdisciplinary collaboration is considered an essential principle underpinning effective primary health care, but translation into practice still seems challenging [18]. In addition, studies have stated that more research is necessary to ensure that the improvement and maintenance of teamwork leads to improved quality and to understand how policy and organisational contexts affect the ability of teams to collaborate effectively as well as how dynamic changes in these contexts influence collaboration within the team [19, 20]. More research is required to understand how policy and organisational contexts affect teams’ ability to collaborate effectively and how dynamic changes in these contexts influence collaboration within a team.

To the best of our knowledge, there is little research on the different contexts that have enacted the trust model and what is experienced as important by FLWs. Furthermore, studies have indicated that there is limited observational data on interdisciplinary practice and that such data could contribute to better understanding the teamwork discourse by identifying elements of interdisciplinary collaborative practice that are not obvious to individuals when asked to self-report [18].

The aim of this study is to explore FLWs’ practices and experiences of working within interdisciplinary teams according to the trust model’s goals.

## Methods

### Study setting

This study was conducted within the home-based healthcare services provided by a large Norwegian municipality that had implemented the trust model. The model recommends that the services should be reorganised into smaller, self-managed interdisciplinary teams in which FLWs are equal members, making decisions through continuous collaboration with each other and service users [7, 8]. The trust model divides home-based healthcare services into several independent, geographically organised interdisciplinary teams [7, 8]. The FLWs within each team meet regularly to discuss user cases and once a week to discuss their collaboration as a team, expectations for work and workload as well as other information that they perceive to be important for sharing in an interdisciplinary environment.

### Study design

A qualitative approach aiming to explore experiences, practices, and phenomena in sociocultural worlds was chosen in this study [21]. Given the limited research on the trust model within community home-based healthcare, the flexibility of qualitative research design was seen as beneficial. It allowed for adjustments congruent with the increased knowledge and insight gained during the study [21]. The data material is based on 37 observations in different kinds of meetings (see Table 1) with six different interdisciplinary teams, 10 individual interviews, and four focus group interviews between March 2021 and April 2022, during the pandemic. The participants were leaders and frontline workers at different levels of the home-based healthcare services, including registered nurses, auxiliary nurses, occupational therapists, physiotherapists, and other unskilled healthcare personnel.

**Table 1:** Overview of the different types of meetings observed

Types of meetings	Number of meetings
Interdisciplinary meetings regarding common users	29
Work meetings within the interdisciplinary teams	3
Leader meetings	3
Profession/ work task meetings	2
Total	37

We started with observations to explore the practice of the trust model and the reorganisation into interdisciplinary teams since it is a method well-suited for developing first-hand knowledge about practice as it is [22]. Due to restrictions related to the pandemic, all the observations were conducted digitally. The first author participated in all of them, while the three other authors alternated as observers. 46 observations were planned, during a two-month period. However, due to unforeseen events as COVID-related challenges, technical difficulties, and the work burden within the home-based healthcare services nine observations were cancelled. Observing digitally made it possible to do almost verbatim transcription of the conversations in the meetings. All four authors took observation notes. For validation, these notes were shared and discussed among the author group.

Individual interviews were then conducted, digitally, aiming for a more in-depth description of the understanding and the performance of the trust model and to challenge the perceptions and interpretations of the observations, 11 individual interviews were conducted. However, one participant withdrew from the study shortly after the interview and before its transcription. Consequently, this data was not included in the analysis, resulting in a final participant count of 10 (see Table 2).

**Table 2:** Participants individual interviews

Profession	IDI Female	IDI Male
Team manager	2	-
Manager assistant	1	-
Registered nurse	1	1
Occupational therapist	1	-
Physiotherapist	1	1
Auxiliary nurse	-	-
Case manager	2	-
Total	8	2

When the focus group interviews were conducted the COVID-19 restrictions allowed for in-person encounters so the interviews were held in meeting rooms provided by the home-based healthcare services. This method was chosen to produce empirical data that can elucidate the norms of group practices and interpretation [22, 23], wishing for the frontline workers to reflect upon and discuss central topics regarding the performance of the trust model. All four groups consisted of an interdisciplinary representation (see Table 3). In three of the groups, everyone was familiar with each other working together as a team, while in the fourth group, some frontline workers did not know all the others. The first author led the focus group interviews while the three other authors alternated.

**Table 3:** Distribution of frontline workers participants in focus group interviews

Profession	Female	Male
Team manager	1	
Manager assistant	1	1
Registered nurse	6	2
Occupational therapist	3	1
Physiotherapist	1	3
Auxiliary nurse	1	1
Case manager	3	1
Total	16	9

All the meetings that were observed and the frontline workers participating in the interviews were recruited through the leaders. The only inclusion criteria were a wish for interdisciplinary representation reflecting the interdisciplinary teams.

The individual and the focus group interviews were semi-structured, using interview guides developed for this study, where themes and some key issues were defined in advance [24].

### Data analysis

The flexibility of qualitative research made it possible to make adjustments to the study based on the insight and knowledge that gradually developed [21]. During the data collection, we discovered similarities in the topics the frontline workers talked about and what we experienced in the observations. The decision was made to merge these data, facilitating a rich and broad analysis. This approach was supported by the fact that the data from the three different methods took the form of conversations rather than field notes. By merging these data, they complemented each other and created a more nuanced and fuller picture [21] Reflexive thematic analysis inspired by Braun and Clarke (2022) was conducted to identify patterns of meaning across the data. The epistemological stance adopted in this study was inspired by social constructivism, with the purpose of understanding the construction of the phenomenon within its social context [25]. The Nordic welfare state provided a particular context, characterised by universalism, home-based care, and state-engineered professionalisation for healthcare workers [26]. The analysis was conducted by employing an inductive approach in which there was no attempt to fit the data into an existing theory; this began with familiarisation of the data and then moved to a systematic coding process, before starting to explore, develop, review and refine themes [27] (see Table 4). Although only the first author wrote and performed the systematic coding and thematising by hand, all four authors contributed to this process through analytical meetings and workshops. Each meeting and discussion led to a deeper and more nuanced understanding of the identified patterns. The themes were developed through collective agreement and collaboration, and they continued to evolve during the process of writing this article. The researchers used their experience from many years in nursing, occupational therapy, and research.

**Table 4:** Example of a few extracts from the analysis

Quote	Codes	Main theme
‘That is really the main point of the trust model, that there should be more user time and fewer employees, there should be recognisable faces for the users. But when there are just hired nurses or many extra shifts,. . then, it would be a little difficult to do the follow-up, at least as a nurse. . .’ (individual interview)	Continuity Resources Patient safety Contentment	**‘We all want the best for service users’**
‘They have these 15 users that they are ‘BOB-responsible’ for and service responsible for,. . that it is a big change. In a way, those who are given the power to find good solutions—those of us who are close to the users, who know the user best—should be able to assist with that. It’s something we’re focused on—many of us—that is, those who have a good relationship with the user can get in touch again to give a sense of security. And creating trust, so that the user trusts us to be able to find good solutions. (focus group interview)	Knowing the users Building trust Tailored services	
‘I feel like I can just pop my head into the purchaser unit employees’ office and ask a question right when I need it, and by doing so, we have solved the case I came in for. The same goes for the rest of the colleagues, whether they are in physiotherapy or occupational therapy or. . You work much more easily with people you’ve seen and know, so that’s a good thing. At least, I feel like we’re working together a lot better.’ (individual interview)	Seeing and talking together regularly is important Knowing each other professionally Collaboration Short pathways for decision-making	**‘Belonging to an interdisciplinary team’**
‘I am more alone with the therapist tasks I have with my service users.’ (individual interview)	Being alone in a professional role within the team	**‘Maintaining belonging to those with similar work tasks and responsibilities’**
‘They say that it is so important to keep purchaser unit employees in the team. . Well,. . maybe we are, but we also need to retreat from the team because we are administering the legislation, and that is something that has not changed after implementing the trust model. We [purchaser unit employees] don’t really have the opportunity to retreat and meet because we are supposed to just be a part of our own IFLT. But the IFLT consists only of people who have not worked with the legislation at all besides me. . So, that is not working. . .’ (observation)	Lack of professional knowledge transition Great responsibility	

## Results

There was diversity among the participants’ experiences of working within interdisciplinary teams and focusing on the needs and wishes of service users and their next of kin as they set out to create flexible and individual tailored services. The results outlined three areas of concern, which are presented in the following themes: ‘We all want the best for service users’, ‘Belonging to an interdisciplinary team’ and ‘Maintaining belonging to those with similar work tasks and responsibilities’.

To maintain the anonymity of the participants, any distinction between the participants and the diverse types of FLWs has been avoided when presenting the results. However, in some sections, differentiations between registered nurses, case managers and therapists have been made solely to highlight certain crucial points and differences between the roles. Quotes have been used selectively to give voice to the participants and to illustrate their meanings. However, in the following presentation they are not connected to the participants to maintain privacy.

### We all want the best for service users

As previously mentioned, the trust model aims to create smaller teams that collaborate with and around service users. The participants in our study were conflicted over whether this aim had been accomplished. Some assumed that service users experienced a positive change regarding the continuity of the FLWs providing their follow-up. One FLW said:

The users must have benefited from the follow-up from one nurse, who is, in turn, responsible for the content of the tasks being delivered. That it is the same person who does both, because I am fairly sure we have accomplished that. (individual interview)

This sentiment was further underscored by another FLW, who expressed that being accountable for 15 users, both in terms of service content and the time that was set for performing the services, brought about significant changes for the FLWs. The goal for the registered nurse was to visit the service user at least once a week and check up on and pay attention to changes in their health and evaluate the service plan. Having smaller teams around the service users was also rated as positive in terms of gaining knowledge and keeping updated about service users:

Now, in a way, we get valuable information about the service users—how things work with and around them and what doesn’t work, right? So, it is positive to be a part of it [the team]. So, I think that benefits service users as well. (focus group interview)

However, other statements showed the vulnerability of smaller teams and, thus, the continuity of the FLWs for service users. This vulnerability was addressed in some meetings by highlighting the challenges experienced regarding the intention to spend more time with service users instead of administrative work and the aim to provide fewer FLWs for service users due to high turnover and sick leave. All participants mentioned in the interviews that this was a threat to the service’s ability to maintain close and continual follow-up of service users and that during a service user’s pathway, there can be several different FLWs from the same profession involved; moreover, not everyone would have in-depth knowledge of service users’ cases.

Regarding doing what was best for service users, another important facet that was mentioned addressed the intention to involve and empower service users in setting goals and to be active participants in their own healthcare service. In both the observations and in the interviews they talked about the importance of asking service users about their wishes, which is a key factor in providing good solutions and services. One FLW said:

If you want to be able to create a good solution around the service user, then they must be involved. The service user’s wishes are important, and if you have not even asked them, then it is not easy to accomplish anything. (focus group interview)

This aim seemed not to be easily achievable due to different reasons, such as economic circumstances, and perhaps, the implementation of the trust model had not changed the opportunities to create a flexible and individually tailored service for service users. This is exemplified by one FLW:

It’s not important what is important for the service user. If we’re conducting an assessment, and even though we’ve suggested all sorts of things, all they want is a place in a nursing home, then the budget comes into play, as it always does. We have to consider whether it’s possible to take care of the service user at home, despite their wishes. And when does it become unsafe, right? So, I do not know if it is that different. (individual interview)

### Belonging to an interdisciplinary team

There were several different views expressed by the participants concerning belonging to an interdisciplinary team. First, the experience of this seemed to be of importance, considering both one’s ability to create flexible and individual tailored services for service users and the experience of one’s own work performance. Having regular weekly interdisciplinary meetings was mentioned by some participants as a new experience. One described in the focus group interview it as being the first time of over 20 years of working within home-based healthcare that they had the feeling of being part of ‘something interdisciplinary’. It was also discussed as the ability to share experiences and knowledge and to be flexible in offering necessary professional roles to service users based on discussions in interdisciplinary meetings. Being together and discussing common service user cases while trying to find a sustainable solution for everyone seemed to be important when it came to the feeling of belonging to an interdisciplinary team. Additionally, some participants experienced a change in the way they communicated with each other. One said:

The biggest change is really having interdisciplinary meetings and sitting close to each other, since that is where you get to discuss the users—that is, where you, in a way, work with the trust model and can discuss the assessments and. . whether we achieve what we are supposed to. . We have a completely different dialogue now. Before, we had a more commando line—‘I decide that you shall. . .’—but now, it is much more like a collaboration. (individual interview)

By getting to know each other better and being accessible and responsive to each other, they seemed to have created a sense of belonging within the team through increased understanding of each other’s tasks, perspectives, and working methods. Hence, they had developed better relationships among the team members and, thus, a kind of trust within the team. One FLW said:

I know the people I work with, they are familiar,. . and that does something for the trust of your colleagues. . However, we could have accomplished that without reorganising the services as well. We are all gathered on the same floor; hence, we are only a short distance away to be able to update each other.

Several of the FLW’s addressed in diverse ways the importance of being located together, despite their professional roles or how the service was organised. The findings indicated that being co-located was of more importance than being co-organised. The participants talked about creating a way of belonging to the team by visiting each other’s offices for short, informal discussions and conducting quick catch-ups on shared service users and cases. Being located together was seen as an advantage enabling these informal and unplanned meetings, and it contributed to individuals’ perception of not being a burden to each other. This seemed to create flexibility and provide better opportunities to find solutions to issues regarding service user cases. This was demonstrated by one FLW, who said:

I feel like I can just pop my head into the case manager’s office and ask a question right when I need it, and by doing so, we solve the case I came to discuss, together. The same goes for the rest of my colleagues, whether they are physiotherapists or occupational therapists. . You work much more easily with people you’ve seen and know, so that’s a good thing. At least, I feel like we’re working together a lot better. (individual interview)

The outcome of closer collaboration, interdisciplinary meetings and the addition of flexibility to service delivery was also addressed by a leader of an interdisciplinary team, who said:

The interdisciplinary collaboration with the occupational therapist and the physiotherapist has started to become more dynamic. They join nurses on home visits when users return home after hospitalisation. Their teamwork appears to be synchronised, particularly in adapting the home environment and circumstances to suit users’ needs. (focus group interview)

Even though the data indicated positive outcomes related to belonging to an interdisciplinary team, this was still seen to be somewhat challenging to achieve in terms of time, attitudes, and habits. One leader said at an interdisciplinary meeting:

We are not isolated poles; we are one organisation, and we need to act that way. From an outsider’s perspective, it seems that we do not communicate with each other. Collaboration over user cases, assessments and decisions; this is something we have to do together. (observation)

### Maintaining belonging to those with similar work tasks and responsibilities

Despite the implication that being organised and located together as interdisciplinary teams had been a positive experience, the participants expressed that it was still important to maintain a belonging with those with similar work tasks and responsibilities. However, this was mostly mentioned by the therapists and case managers. One case manager said:

And they say that it is so important to keep the case manager in the interdisciplinary team. Maybe it is, but we also need to retreat from the team because we are administering the legislation, and that is something that has not changed after implementing the trust model. . We don’t really have the opportunity to retreat and meet each other, since we are supposed to just be part of our own interdisciplinary team. But the team consists only of people who have not worked with the legislation at all, besides me. . So, that is not working. . (observation)

The therapists also discussed in one meeting the implications of feeling compelled to relinquish their professional affiliations, which left them feeling alone regarding their professional responsibilities when making assessments and handling their tasks within the team. Additionally, they conveyed their uncertainty about whom to reach out to, such as when they needed to transfer some of their cases or share knowledge to find suitable and effective solutions for complex situations. One therapist said:

There is no one with whom you can discuss solving things or when to put your foot down in a service user case. For the leader, it is a completely unknown service user and professional role, so he cannot help. So, it is not just as simple as to always involve management. . We need a professional forum where we can discuss and exchange experiences and knowledge—that is important. But we are good at visiting each other’s offices. . There is a lot of good knowledge among us, but we are not even sitting on the same floor, so it makes things a bit difficult. (individual interview)

Feelings of being pressed for time when it came to tasks waiting to be assessed or being alone with the decisions to be made were mentioned by both the therapists and the case managers both in a meeting and the interviews. They talked about these as negative feelings, seemingly giving root to a sense of being unsatisfied with their own work situation. This was raised by a case manager, who said:

I don’t know if they think about that all the 140 service users per team need a decision made about home-based healthcare, or they should perhaps have an assessment about practical assistance or something. And all those decisions should eventually be re-evaluated. . You’re quite alone in a lot of things—when dealing with heavy cases and with next of kin who are angry if they do not get their way. Before, I felt valued; I felt that I was significant to the organisation; I felt that my opinions counted—the ideas I suggested—that everything was, in a way, valuable,. . but I do not feel that way anymore. . (individual interview)

Maintaining belonging and collaborating with those with the same tasks and responsibilities were things the therapists and the case managers strived for, and they tried to find solutions, despite the co-organisation into interdisciplinary teams. One occupational therapist said:

When we had Thursday meetings, we felt it gave us a good foundation for our work. . However, the management felt like we were trying to cling to old things that we weren’t supposed to hold onto. So, we were told to quit those meetings; we were supposed to belong only to the interdisciplinary teams. But when we had those weekly meetings, we helped each other prioritise, and we discussed cases. . They were very useful meetings. We’re trying to sneak the meetings back in. So, this summer, we’re going to have, like, five minutes here and there. (individual interview)

The data indicated that the organisational change from being co-organised was experienced as demanding and appeared to be an inhibitor of the trust model’s goal to create well-functioning interdisciplinary teams. Apparently, these experiences were things the therapist and the case manager had even treated as unwanted before the co-organisation entered into force, addressing it as a topic for discussion with the management ahead of the implementation. This concern was also related to personnel resources, especially regarding sick leave and vacations. One therapist said:

We also suggested at the time we co-organised that perhaps the occupational therapists, physiotherapists, and case manager could be in a kind of separate team in which we could have the main responsibility for the different teams, but one where we then had a unified leader who could also use us in different places when there was sick leave or vacation or situations like that. (observation)

On the other hand, the leaders also addressed these experiences and attitudes among some of the employees, understanding them to be a form of resistance within the interdisciplinary team. One leader said of the therapists and case managers:

They should see themselves as part of the daily workforce. They have to be more present with the team, demonstrate that they are a part of the team and those things. . Then, I believe the understanding of the roles within the team and the collaboration would be better. It is not ‘them and us’; we are a team, together. (observation)

## Discussion

This article examines the FLWs experiences and practices within the trust model’s goal of working within interdisciplinary teams. In this section, we discuss the findings related to how the FLWs enacted the trust model’s goal and how this might influence their ability to empower competence and knowledge in their work. Lipsky (2010, p. xxii) stated that to understand how and why performance contrary to intentions—and, to some degree, regulations—happens, it is important to understand how the rules are experienced by the workers in organisations, what latitude they have in acting on their preferences and what other pressures they experience. Our findings address this and outline conflicting experiences in distinguishing between the trust model’s intention for interdisciplinary teams, the way this was carried out in the organisation and how it was practiced. The findings suggest a dilemma between the expectation of creating social belonging to a team and identifying as a member of an interdisciplinary team while, at the same time finding a way of maintaining belonging to those with the same tasks and responsibilities.

### Creating an identity and social belonging as an interdisciplinary team member

The trust model was implemented with the intention of increasing interdisciplinary teamwork and the goal of providing more flexible and individually tailored services through smaller interdisciplinary teams with common goals and understanding of service users’ needs and service delivery [7]. Understanding of professional roles, trust and communication is an important factor in interdisciplinary practice [28]. Co-organising or working within an interdisciplinary team is seen as an incentive to bring FLWs together to achieve more efficient interdisciplinary services [29]. The findings indicate that the FLWs aimed to foster team unity by collaborating, setting common goals and sharing decision-making. These goals aimed to create flexible and tailored services for service users, which was the clear expectation of the leaders. It is a general challenge that FLWs do not sufficiently know each other’s areas of work [10, 30, 31]. Hence, they might have incorrect or unrealistic knowledge and expectations of what others within their teams should and can contribute to, and achieving this knowledge takes time [31, 32]. The participants in our study experienced beneficial outcomes from teamwork, which seemed to enhance collaboration, decision-making and knowledge translation within the team.

In implementing a trust model, FLWs deal with and negotiate new standards for executing their work, which can influence their professionalism [33, 34]. These new standards, for instance, involve less focus on collaboration as well as professional discussions and development with colleagues within one’s own profession or with those handling the same tasks and responsibilities; there are new managers, new roles and dynamics within the team and new office settings [33]. These new standards are set by public policies and leaders, and the literature shows that policy objectives have a tendency to not always be followed due to FLWs’ solidarity, which lies with service users’ needs and their own professional standards [33, 35]. Our findings point to a common wish among FLWs to do good for service users and be able to deliver high-quality interdisciplinary services that focus on what matters to service users, which could be understood as an example of solidarity for both service users and professional standards.

Working together as an interdisciplinary team can create a better user pathway due to collaboration within the team, making it possible to be flexible in service delivery, provide quicker responses by each professional within the team and have closer relations between the team members and with the service users [7, 31]. This study highlights these aspects, understood as parts required to accomplish the trust model’s goal. Another aspect that was often mentioned concerns how co-localisation led to more informal meetings and, thus, both easier and faster shared decision-making. These findings are supported by research showing the importance of being able to mobilise and coordinate FLWs’ resources when they are needed [10]. Being part of an interdisciplinary team allows FLWs with different educational backgrounds and task expertise to come together, pooling their knowledge, competence and skills to address complex service user cases. Our findings indicate that the FLWs could leverage their unique perspectives and approaches to provide comprehensive and integrated care. It can further be understood that this interdisciplinary dynamic also fosters innovation, since the FLWs learned from each another and developed a deeper understanding of each other’s roles [36]. By doing so, the participants felt connected to the interdisciplinary team, and some mentioned a feeling of performing interdisciplinary work for the first time in their careers.

### The importance of peers

The findings show that despite their enjoyment of working more closely within the interdisciplinary team, some FLWs also needed to be connected to those with similar work tasks and responsibilities. The therapists and the case managers highlighted, in different settings, these two sides of being organised into interdisciplinary teams. The need to still be able to discuss tasks and legalisation and share knowledge, competence, and experiences with those with similar work tasks and responsibilities was raised. Lipsky (2010) highlighted the significance of FLWs’ discretion and autonomy in decision-making as they navigate complex situations and adapt policies to fit individual cases. Despite the organisational form and sense of belonging, the case managers worked closely with legislation and thus had overarching responsibility for prioritising the allocation of services. This responsibility and work task demands discretion and autonomy when being practiced - and sufficient trust in the capacities of the case managers to do so [33]. The case managers in our study talked about the need for space to work closer together than the trust model had intended and mentioned that they felt their work was underestimated as they implemented the trust model. Some also mentioned feeling a lack of trust and appreciation after being organised into interdisciplinary teams.

The importance of the possibility of having professional discussions with those with a similar educational background was mentioned by the therapists. Being able to empower each other by sharing knowledge and competences and discussing tasks and solutions was missed. This could indicate that it is necessary to keep a connection with those of the same profession to maintain and develop one’s professional identity and to empower professional discretion and autonomy [37], thereby resulting in self-confidence in decision-making, particularly when dealing with complex user cases. Furthermore, the importance of maintaining belonging to a professional identity was also outlined as a premise for providing sufficient, flexible and individually tailored services. This understanding is supported by other studies showing that continuing professional development raises professional standards through the competences gained from this, thereby increasing professional performance with positive benefits for patients, organisations and individual healthcare workers [37, 38]. It could also be important for enacting the responsibility of delivering equal services. By not being able to discuss cases and responsibilities with peers, the case managers and therapists seemed to be impeded from having an overarching overview of assessments based on needs and rights, thereby basing their decisions more on isolated individual discretion instead of a coherent and equal approach to service user cases.

Contrary to the therapists and the case managers, the registered nurses and other FLWs in our study did not mention this loss of connection and collaboration with peers. This indicates that they still had the opportunity to discuss matters and be involved in and evaluate tasks and responsibilities. Even though there may only be a few nurses on each shift, they have a constant relationship with the rest of the care staff delivering the same tasks on their worklists [39], which enables them to collaborate in assessing and evaluating the service user cases and the tasks given. The second argument for claiming that this is different for this grouping within an interdisciplinary team is that they share experiences, knowledge and competences on a daily basis during work shift meetings and through nursing care plans related to their worklists. Therefore, the dilemma of dual belonging seems to have been mostly related to therapists and case managers and points to a negotiation that can be understood as a way of gaining control of one’s own work situation and creating meaning in a new organisational and work structure (33).

The trust model’s intention seemingly does not embrace the dilemma of dual belonging when encouraging organisation into interdisciplinary teams. Our findings indicate that the significance of engaging in dialogue within one’s profession or with those with similar tasks and responsibilities might have been underestimated. The focus seems to be on creating belonging to the interdisciplinary team rather than acknowledging the strengths and synergies that evolve when belonging among and collaborating with peers. A study focusing on evidence-based practice in primary healthcare showed that due to an organisational and managerial focus on efficiency rather than on quality of care, there are few or no incentives for promoting the professional development necessary for FLWs [40], which might also seem to be happening in the trust model. Studies have shown the importance of organisation into interdisciplinary teams to create healthcare services that are both better and sustainable, and they have stated that it is a protracted process of development to learn about the different competencies within a team and to see the differences as an advantage rather than a problem [41]. However, in the trust model, there seems to be less focus on the professional diminution that follows. This study implicates vulnerability, especially among the therapists and the case managers, leading to situations that could eventually threaten user safety due to there being less opportunity to develop and discuss common tasks and cases. The outcome of this is that there is less competence in solving cases because of there being fewer professional colleagues with whom one can discuss cases and higher vulnerability related to sick leave because of the dwindling number of members of each profession represented within the team, which seems to be a risk. These findings indicate a need to facilitate and therefore maintain an arena for professional groups or for groups with similar work tasks and responsibilities to get together despite the organisational form. Furthermore, this leads to a potential discussion when it comes to striving for co-organising into interdisciplinary teams when co-location seems to be more accepted by FLWs and crucial in interdisciplinary work. When striving for a more coherent and interdisciplinary healthcare service, interdisciplinary teams and co-organisation are some means to accomplish this [29]. More co-creation, flexibility and collaboration and a sustainable home-based healthcare service seem to be the main goals [42], and it is worth questioning whether it is the co-organisation or the co-location that contributes most to achieving them. On the other hand, building relations based on trust is a long-term process that could be difficult to achieve when FLWs belong to different organisations [43]. To take an approach that takes advantage of professional and interdisciplinary skills and discretion when providing flexible and individually tailored services can be a way of building this trust-based relationship. This supports the focus being on relationships and actions rather than on organisational structures (33, 43).

### Limitations

The key limitations of this study relate to the generalisability of the findings. Even with the large amount of data gathered using several methods, the findings of this study were derived from the single municipality that was the subject of our analysis, and generalisability to other settings may be constrained. Nevertheless, we have strived to make it possible to transfer the findings to other settings with similar contexts by describing the context as descriptively as possible. By describing the process of analysis and including quotes in the results section, we have tried to make our analysis transparent [27]. Additionally, there are several other aspects related to interdisciplinary teamwork, and to the trust model that might have been relevant to address in this study. Our choice was rooted in the needs-led research process this project has followed [15]; however, by using different theories or conducting data from other settings.

could have provided another discussion. Even though this study provides insights into the experiences of being organised into an interdisciplinary team within a home-based healthcare context, the results cannot be broadly generalised.

## Conclusion

This study demonstrated how FLWs enacted a trust model’s expectation of working within interdisciplinary teams. The findings demonstrate that the FLWs experienced a dilemma between creating belonging to and forming identities within the interdisciplinary team, and at the same time, they addressed the importance of maintaining belonging and identity with those in the same profession or with the same tasks and responsibilities. This seems to be underestimated within the trust model, and by acknowledging this, organisations and policymakers can create environments that supports both. This can enhance FLWs’ possibility to deliver flexible and individually tailored services for service users.

This study has communicated valuable knowledge, making it possible to illustrate some aspects of the trust model in a Norwegian home-based healthcare context. Demographic changes are forcing healthcare services to restructure and implement innovative solutions. Further research is needed to investigate interdisciplinary work and organisational work structures to better understand the prerequisites for interdisciplinary collaboration in home-based healthcare. This will provide valuable knowledge to the ongoing discussion on how to bridge interprofessional competence, collaboration, user needs and resources into effective, high-quality, flexible and individually tailored services. This study provides insights and avenues for consideration by politicians, leaders, FLWs, researchers and others interested in interdisciplinary front-line teams in home-based healthcare.

### Electronic supplementary material

Below is the link to the electronic supplementary material.

Supplementary Material 1

Supplementary Material 2

## Data Availability

The dataset is derived from observations and interviews, all in Norwegian, and is not publicly available because of the terms of the data collection approval. However, parts of the data can be made available by the corresponding author upon reasonable request.

## Abbreviations

FLWs: Frontline workers
