# Supplementary material for: Balancing between dual belongings when organised into interdisciplinary teams, with the trust model as the context: A qualitative study
Source: BMC Prim Care. 2024 Aug 24;25:314. doi: 10.1186/s12875-024-02554-7 (PMC11344355; doi:10.1186/s12875-024-02554-7)
Supplement: Supplementary file 1 — Supplementary Material 1 [file 12875_2024_2554_MOESM1_ESM.pdf]

Intro:

I'm glad you had the opportunity to have a short conversation/interview with me. I have some questions following the observations I've made, but they're not about specific client cases or situations I've observed. More like curiosities that I want to discuss with you. I'm going to limit the interview to 20 minutes, so I might have to stop us in the middle of a question to move on to the next one - just to make sure we get through the three questions I've prepared. It might go quicker too, but if so, that's not a problem.

The conversation will be recorded, I'll let you know when I start the recording. So I must remind you not to mention any names, neither of clients nor colleagues. Do you have any questions about the information or the interview?

I'm going to start the interview by addressing consent, just like I did in each observation. I will then use the consent text that is in the information letter that you have received in advance.

Audio recording from here:

- Have you received and understood information about the study where we are now going to do an individual interview? And have you had the opportunity to ask questions?
- Do you consent to the interview being recorded and then transcribed anonymously for further use as data material?
- I also need to inform you and need your consent that you know that your consent can be withdrawn at any time and then the audio recording and the linkage key I have made on your name will be deleted.

Anonymized data, i.e., the transcription of this interview - anonymously - will still be part of the data material, but it is in no way possible to obtain information that links it to you - or find out through the material that you have said this.

The basis for the study, or the frame around it, is the trust Model, and how you practice the "tailoring" of services around your users - that is, the intention of flexible and individually tailored services.

**Interview Guide**	**Follow-up interview after observation.**
**Purpose**	The observations require some clarifications and a deeper understanding of how the trust model contributes to flexible and individually tailored services.
Who	Administration manager, physio/ergo HVR, case managers, service responsible nurse, and coordinating nurse.
Time per interview	20 minutes
Location	Zoom (encrypted and validated as secure for research).<br>Audio recordings are made and sent to a secure area on the researcher's domain.<br>.
**Questions**	
1.	What would you say contributes to your ability to offer individually tailored and flexible services to the users?
2.	What would you say are the changes in collaboration around the users after you implemented the trust model?<br>• Meeting structure? New meeting forums? Forms of interaction?<br>• Decisions; how/who makes, adapts, and concludes them?<br>New organization? Work teams?
3.	What do you think are the success criteria necessary for you to provide individually tailored and flexible services?
