# Supplementary material for: Balancing between dual belongings when organised into interdisciplinary teams, with the trust model as the context: A qualitative study
Source: BMC Prim Care. 2024 Aug 24;25:314. doi: 10.1186/s12875-024-02554-7 (PMC11344355; doi:10.1186/s12875-024-02554-7)
Supplement: Supplementary file 2 — Supplementary Material 2 [file 12875_2024_2554_MOESM2_ESM.pdf]

**Interview guide**	Focus group interviews
Aim	How do employees in the service reflect on the concept of "flexible and individually tailored home-based services", considering national health policy guidelines, professional roles, and guidelines for the trust model?
Who	4 groups of 5-7 employees; physiotherapist, occupational therapist, case-managers, service responsible nurse, coordinating nurse and healthcare workers (unskilled).
Time	75 minutes
Location	Physically at the workplace of the employees
**Questions**	
1.	**We are here today to talk about the Trust Model, and what we have chosen to call "tailoring" of services- which is about how you create flexible and tailored services for individual users. I want us to start by talking about the Trust Model. What do you know about it, or what significance does it have in your daily work?**
2.	**Can you tell us a little about how you collaborate to be able to offer services that are adapted to the individual user?**<br>• How are users referred to you?<br>• How do you collaborate on mapping the user's situation/needs?<br>• How do you interact about the user?<br>o Written referrals? Phone calls? Via journal systems? In the hallway? Formal/informal?<br>• How do you work to bring out what is important for the user and how do you use the answers/goals in your collaboration?<br>• What does cross-disciplinary/team/department collaboration mean to you?<br>• What are important factors for achieving good collaboration? Do you have examples?
3.	**Empowered employees and professional discretion are a goal in the trust model. What do you think are characteristics of this? Do you have examples of feeling or not feeling empowered?**<br>**Are there any specific situations where you experience this to a greater or lesser extent?**<br>• How can it be facilitated for you to experience this?<br>• And what do you perceive as obstacles for your professional discretion?<br>• An intention with this professional discretion is that one should be able to be close to the user to quickly be able to detect changes in needs and then be able to adapt the service continuously after that. How do you experience this professional discretion? Examples?<br>• Examples of empowered employees?
4.	**Smaller teams, with fewer employees for the user to relate to, should be a measure in the trust model to achieve this individual adaptation of the services to the users. How do you experience this?**

	• What does flexible and individually tailored services mean to you? What does it mean to tailor the services for your users?<br>• Do you feel you know the users well, and are you able to keep up with changes in the user in an appropriate way?<br>• Has your collaboration changed after this adaptation, and in what way?<br>• What is needed to be able to offer this follow-up and continuity with the users?<br>• How do you perceive that you are using each other's resources strengths in the teamwork?

4.
